# Individualized frequency and montage tACS to engage theta-gamma coupling and enhance working memory in mild cognitive impairment

**DOI:** 10.3389/fpsyt.2025.1565881

**Published:** 2025-06-02

**Authors:** Mina Mirjalili, Iryna S. Palamarchuk, Heather Brooks, Reza Zomorrodi, Ashley Melichercik, Wei Wang, Sean M. Nestor, Daniel M. Blumberger, Abhishek Datta, Christopher Bowie, Bernadette Mdawar, Kullervo Hynynen, Sanjeev Kumar, Tarek K. Rajji

**Affiliations:** ^1^ Campbell Family Mental Health Research Institute, Centre for Addiction and Mental Health, Toronto, ON, Canada; ^2^ Adult Neurodevelopment and Geriatric Psychiatry Division, Centre for Addiction and Mental Health, Toronto, ON, Canada; ^3^ Department of Psychiatry, Temerty Faculty of Medicine, University of Toronto, Toronto, ON, Canada; ^4^ Temerty Centre for Therapeutic Brain Intervention, Centre for Addiction and Mental Health, Toronto, ON, Canada; ^5^ Biostatistics Core, Centre for Addiction and Mental Health, Toronto, ON, Canada; ^6^ College of Public Health, University of South Florida, Tampa, FL, United States; ^7^ Harquial Centre for Neuromodulation, Sunnybrook Health Sciences Centre, Toronto, ON, Canada; ^8^ Research and Development, Soterix Medical Inc., Woodbridge, NJ, United States; ^9^ Department of Psychology, Queen’s University, Kingston, ON, Canada; ^10^ Department of Medical Biophysics, University of Toronto, Toronto, ON, Canada; ^11^ Physical Sciences Platform, Sunnybrook Research Institute, Toronto, ON, Canada; ^12^ Toronto Dementia Research Alliance, University of Toronto, Toronto, ON, Canada; ^13^ Department of Psychiatry, O’Donnell Brain Institute, University of Texas Southwestern Medical Center, Dallas, TX, United States

**Keywords:** mild cognitive impairment, theta-gamma coupling, individualization, tACS, working memory

## Abstract

Mild Cognitive Impairment (MCI) is a clinical prodromal stage of Alzheimer’s disease. Enhancing executive functions in patients with MCI could optimize cognitive compensatory mechanisms and slow cognitive decline. The prefrontal cortex (PFC) and its connections to the hippocampus support executive functions, including working memory. Transcranial alternating current stimulation (tACS) can modulate these connections by engaging theta-gamma coupling (TGC) and may thereby strengthen working memory. This study, “tACS to engage theta-gamma coupling and enhance working memory in MCI” (tACS-MCI), will assess the feasibility and cognitive effects of EEG and MRI-guided individualized tACS. The stimulation will target the prefrontal and temporal cortices in 20 MCI participants. Participants will be randomized to receive either individualized tACS or sham tACS for 10 days. tACS individualization will involve adjusting the theta frequency, tACS electrode locations, and current intensity for each participant. Cognitive and functional assessments will occur at baseline and post-intervention. We aim to determine: 1) the feasibility of individualized tACS in MCI, including recruitment and retention; 2) whether tACS engages TGC by assessing its increase in response to tACS; and 3) changes in N-back working memory performance following tACS, as well as whether changes in TGC mediate the changes in performance. The tACS-MCI study will employ an EEG and MRI-guided individualized approach to promote synchronization between frontal and temporal cortices, using participant’s unique brain structure and neurophysiology. We aim to assess the feasibility of this novel intervention as a potential approach to more effectively prevent cognitive decline.

## Introduction

Dementia is a progressive loss of cognitive and behavioral abilities that significantly impacts quality of life. Alzheimer’s disease (AD) is the most common cause of dementia among older adults (1). However, by the time AD symptoms become evident it may be too late to slow progression of the neurodegeneration. Thus, identifying effective strategies to prevent and slow the progression of AD is crucial.

For prevention and early intervention in AD, we propose this study that is focused on Mild Cognitive Impairment (MCI), i.e., the clinical prodromal stage (2). Specifically, in MCI, the prefrontal cortex (PFC) enables compensatory mechanisms that could delay progression to AD (3, 4) via executive functioning (5, 6). MCI patients with higher executive functions perform better on verbal memory than those with low executive functions (3, 7). MCI patients’ verbal memory performance is also associated with PFC thickness beyond any association with the temporal cortex (3). Impairments in executive function (4, 8) and working memory (9, 10) drive progression from MCI to AD. Thus, interventions that enhance executive function and working memory could prevent or delay progression from MCI to AD.

Executive functions in general and working memory in particular are supported by the PFC and its connections to other parts of the brain, including hippocampus and temporal cortex (11–13). Among these connections, those connecting the PFC to the hippocampus either directly or through intermediary regions are critical (14). The role of the PFC-hippocampus connections has been established in several studies where disconnecting the PFC and the hippocampus in rodents results in working memory deficits (15–20). Working memory refers to the ability to manipulate and maintain items of information within a short period of time (21, 22). In our heuristic model, neuronal assemblies generating gamma oscillations represent these items of information (23). Further, the hippocampus entrains PFC theta oscillations (24, 25), causing synchrony between the hippocampus and PFC. This synchrony drives the order of activation of the neuronal assemblies, and thus, provides the temporal context for the PFC to manipulate information (26–28). Using electroencephalography (EEG), this process results in the coupling of the amplitude of gamma oscillations to the phase of theta oscillations (theta-gamma coupling, TGC) during a working memory task (29, 30).

Preclinical (27, 31) and clinical neurophysiology studies, including those in MCI and AD (30, 32–34) show that modulation of TGC supports executive functions, particularly working memory. A study using intracranial EEG in humans with epilepsy has also shown that frontal and hippocampal activity are coordinated via TGC during working memory (34). Accordingly, our study aims at enhancing TGC to support working memory in MCI. One approach to enhance TGC is using transcranial alternate current stimulation (tACS). tACS has been shown to engage TGC and, through this engagement, enhance working memory in healthy older adults (35). In that study, a high-definition tACS system with an M × N nine-channel configuration was utilized. Target areas included the left PFC and the left temporal cortex. The alternating current was in-phase across the two targeted regions (i.e., 0° relative phase difference) to promote network synchronization. While participant-specific theta frequency was applied for each participant (“frequency-individualized stimulation”), stimulation intensity and electrode montage remained non-individualized and a generic brain MRI was used to optimize electrode placement across participants (“non-individualized optimized stimulation”) (35). However, given anatomical variability across participants specifically in MCI participants with potential brain structural atrophy, this frequency-only individualization may not achieve the required electrical field and sufficient dosing, for each individual target to elicit measurable neurophysiological effects (36–38).

Given the high inter-individual variability in tACS response documented in the literature, we developed a fully individualized approach. Utilizing individual EEG and structural MRI data, we will determine individualized theta frequency for delivering tACS to enhance synchronization between PFC and temporal cortices using individualized electrode montage (i.e., electrode locations and their currents based on each participant’s unique brain MRI) to ensure that the maximum electric field reaches the specified targets (“EEG and MRI-guided individualized stimulation”). We will then deliver this EEG and MRI-guided individualized tACS bilaterally, considering that compensatory mechanisms involve both the left and right PFC (6). There is also evidence of neural modulation by tACS when applied bilaterally (39, 40). We believe that this study will be instrumental in furthering potential preventive strategies for cognitive decline in MCI, as no study to date has assessed the effect of EEG and MRI-guided individualized tACS on TGC and cognitive function in AD or MCI.

## Design

### Overview

We propose to investigate the feasibility and preliminary effects of EEG and MRI-guided individualized tACS on TGC and working memory in MCI participants. This proof-of-concept study will test whether tACS engage TGC as a target and, in turn, enhance working memory. We aim to (1): determine the feasibility of tACS in older individuals with MCI and examine recruitment and retention (Objective 1) (2); determine TGC engagement in response to tACS by assessing whether TGC increases in response to tACS during a working memory task (Objective 2); and (3) assess change in working memory in response to tACS, and whether changes in TGC mediate changes in working memory performance (Objective 3).

We hypothesize that at least 30% of screened participants will agree and be eligible to receive the intervention they are assigned to (Hypothesis 1a (H1a)); at least 70% of participants will attend at least 80% of their treatment sessions (H1b); participants randomized to tACS will experience higher increase in TGC than those randomized to sham-tACS (H2); participants randomized to tACS will experience more improvement on working memory from baseline following the intervention than those randomized to sham-tACS (H3a); and across all participants, change in TGC will mediate change in working memory performance (H3b).

**Figure 1:**
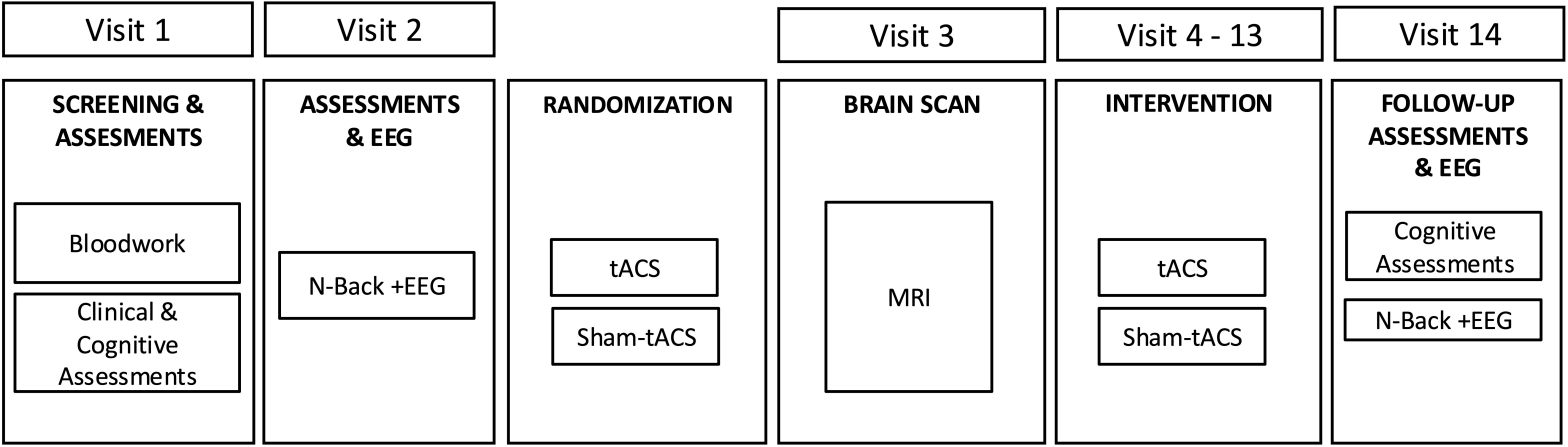
Overall study design for tACS and Sham-tACS groups.

We will randomize 20 MCI participants to receive either EEG and MRI-guided individualized tACS or sham-tACS (1:1). Sequential bilateral tACS or sham-tACS will be delivered to the PFC and temporal cortices. Each participant will receive daily stimulation for 5 days per week for two weeks. Working memory and TGC during working memory performance will be assessed at baseline and after the last intervention session. Working memory will be assessed using the N-back task (41) with 2-back d’ index being the primary measure as justified by our preliminary findings (30, 32, 42). TGC will be assessed using EEG during N-back performance. **Figure 1** represents the overall study design.

### Participants

#### Participants will meet the following inclusion criteria

1) Age 60 years or above, 2) Diagnosis of MCI due to AD using the core clinical criteria by the National Institute on Aging and Alzheimer’s Association for MCI participants (NIA-AA) (43) and ascertained by a study investigator, 3) Objective evidence of single or multi domain MCI using a comprehensive neuropsychological battery, 4) willingness to provide informed consent, 5) ability to read and communicate in English (with corrected vision and hearing, if needed).

#### Participants will also be excluded if they meet the following exclusion criteria

1) Current use of an acetylcholine esterase inhibitor or memantine, 2) Major Depressive Disorder with active symptoms in the last 3 months, 3) a lifetime diagnosis of bipolar disorder; intellectual disability; or a psychotic disorder, 4) substance use disorder active in the last 3 months, 5) any other DSM-5 (44) diagnosis that may be associated with prefrontal cortical dysfunction as ascertained using the study investigator opinion, 6) current anticonvulsant use due to its impact on brain stimulation induced activity. An exception will be made if they are taking gabapentin or pregabalin AND if the dose had been stable for at least 4 weeks prior to study entry AND if prescribed for chronic pain, 7) current benzodiazepine use of more than what is equivalent to lorazepam 2 mg/day. This is due to their known pro-GABAergic activity and the suppressive effect of GABAergic agents on cortical plasticity, and 8) any contraindication to MRI or contraindication to tACS (e.g., cardiac pacemaker, acoustic device, history of seizures) (45, 46).

### Assessments and outcome measures

#### Baseline assessments

##### Clinical and cognitive assessments

###### Participants will undergo the following assessments

Clinical: MINI International Neuropsychiatric Interview (MINI) (47) or Structured Clinical Interview for DSM-5 (SCID) (48) to ascertain eligibility, and the Clinical Dementia Rating Scale (49) (CDR) to assess current functional status. Participants will also be asked to provide results from clinical blood tests undertaken within the previous 6 months or undertake new lab tests prior to commencing the study. The required lab tests will be complete blood count (CBC), Sodium, Potassium, Chloride, Bicarbonate, Urea, Creatinine, Alanine Aminotransferase (ALT), Aspartate Aminotransferase (AST), Alkaline Phosphatase (ALP), Gamma-Glutamyl Transferase (GGT), Cholesterol, High-Density Lipoprotein (HDL), Thyroid-Stimulating Hormone (TSH), and Vitamin B12, as these could be related to aging and cognitive decline.

Cognitive (other than N-back): Participants will be tested using the Montreal Cognitive Assessment (MoCA) (50) to ascertain eligibility and the Wide Range Achievement Test-4 (WRAT) Reading Recognition Subtest (51) to estimate premorbid IQ for the interpretation of the cognitive scores. We will also use a neuropsychological battery that is well-established, standardized, reliable, and well-tolerated by older adults with various brain disorders, including MCI as reported in our recent publication (30, 52), and is also used in two ongoing clinical trials in MCI by our team (NCT02386670; NCT04583215). We will assess the following domains with the following tests: Attention (Continuous Performance Task – Identical Pairs (53)); Language (Boston Naming Test (54), Letter & Category Fluency (55)); Executive Function (Trail Making Test Par B (56), Delis-Kaplan Executive Function System (D-KEFS): Color-word interference (57); Clock Drawing (58))*;* Visuospatial Functioning (Benton Judgement of Line Orientation (59)); Visuospatial Memory (Brief Visuospatial Memory Test-Revised (60)); Processing Speed (Trail Making Test Part A (56), WAIS-IV Coding Subtest (61)); Verbal Learning and Memory (California Verbal Learning Test (62)); and Working Memory (Letter-Number Sequencing (63)). The battery takes about 2 hours to complete and will be administered on Day 1 as part of the baseline assessments. Participants’ performance on the battery will also be used to confirm eligibility and the type of MCI. For each domain, a composite z-score will be generated using performance on each individual test within the domain. The NP tests will be repeated at the follow-up visit to evaluate cognitive performance before and after the brain intervention. **Table 1** represents the outcome measures from the neuropsychological battery.

**Table 1:** The outcome measures of neuropsychological battery.

Cognitive Domain	Test	Measure
Verbal Learning & Memory	California Verbal Learning Test Second Edition (CVLT-II) (62)	Total correct responses for trials 1–5 Percent retained at long delay trial from trial 5 at immediate recall condition d’ Hits and False Alarms of recognition Yes/No responses
Processing Speed	Trail Making Test Part A (TMT-A) (56)	Seconds per correct connections
	WAIS-IV Coding subtest (61)	Total correct responses
Visuospatial Memory	Brief Visuospatial Memory Test-Revised (60)	Total correct responses for trials 1–3 Percent retained on delayed recall trial
Executive Function	Trail Making Test Part B (56)	Ratio of seconds per correct connections for Trail Making Test Part B over Part A or B minus A
	Delis-Kaplan Executive Function System (D-KEFS): Color-Word Interference (57)	Average of “Inhibition Vs. Color Naming S.S.”, “Inhibition/Switching vs. Combined Naming + Reading S.S.” and “Inhibition/Switching vs. Inhibition S.S.” or Total Time to Complete for Condition 3 and Condition 4
	Clock Drawing Test (58)^110^	Total Score
Attention	Continuous Performance Task – Identical Pairs (CPT-IP) (53)	d’ across all three trials
Language	Letter & Category Fluency (55)	Total Score
	Boston Naming Test (BNT) (54)	Total spontaneous correct responses and correct responses after a stimulus cue
Working Memory	Letter-Number Sequencing (LNS) (63)	Total correct.
Visuospatial functioning	Benton Judgement of Line Orientation^109^	MOANS age/education corrected scaled score

**Figure 2:**
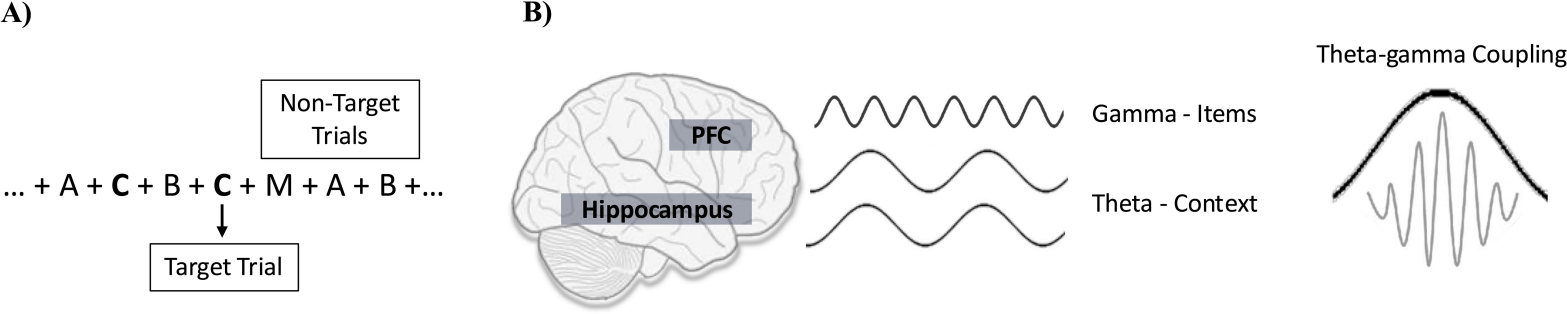
N-back task (2-back) and TGC. **(A)** 2-back task: To respond correctly, the participant must hold in memory all recent letters which are represented by gamma oscillations in the PFC. In addition, the participant has to hold these letters “in mind” in the correct order. The correct order depends on the N-back condition which determines the time span (1, 2, or 3 letters back) over which the order needs to be maintained. It also depends on the new letter that is presented because every new letter triggers the updating of the order since it needs to be added to the list of the recently presented ones. **(B)** TGC: During the N-back task, participants are required to recall the sequential order of letters presented on the screen. Each letter is represented by a distinct pattern of neuronal activation within the prefrontal cortex (PFC), resulting in unique gamma oscillations. These neuronal assemblies are linked to a larger assembly spanning PFC-hippocampus connections, which encodes the order of letter presentation. This synchronized activation couples gamma oscillations to specific phases of theta oscillations, known as Theta-Gamma Coupling (TGC). Ultimately, TGC contribute to accurate recollection of letter sequences.

The N-back task: Following the baseline assessments, participants will undergo an N-back-EEG as we have done previously (64). During the N-back the participant is presented continuously on a computer screen with a series of letters, one at a time. For every letter, the participant has to determine whether this specific letter matches (i.e., a target letter) or not (i.e., a non-target letter) the letter that was presented N trials back, with N being 1, 2 or 3 depending on the session (**Figure 2A**). The N-back task assesses working memory capacity, maintenance and manipulation of information and is sensitive to PFC dysfunction (41). It has good test-retest reliability and minimal practice effects (65), which makes it suitable to assess the effect of the intervention.

The N-back-EEG will then be repeated after the 10-session course. The primary outcome measures will be based on the 2-back condition as justified by our preliminary findings (30, 32, 42). The N-back accuracy will be assessed using *d’*, a sensitivity index based on the z scores of the hit and false alarm rates using the following formula: *d*’ = z(H)- z(FA) where H is the hit rate and FA is the false alarm rate. We will use this index for randomization to active vs sham (see below).

Measuring TGC: EEG data will be collected using a 64-channel Synamps 2 EEG system and the 10–20 montage system while participants complete the N-back task. The reference electrode will be placed posterior to Cz. The sampling rate for collecting EEG data will be 1 kHz and we will apply DC and low pass filter of 100 Hz to the signal. Then we will perform preprocessing using MATLAB (The MathWorks, Inc.) and EEGLAB following established methodologies (29, 30, 32). To calculate TGC, we will first extract theta (4–7 Hz) and gamma (30–50 Hz) oscillations using second-order zero-phase shift filters. We then calculate theta phase and gamma amplitude using Hilbert transform. Since a longer signal ensures the reliability and stability of the TGC value, we will concatenate the epochs to reach to a signal of 5,000 ± 150 ms (29). We will then compute TGC by segmenting theta phases into eighteen 20° intervals and constructing a phase-amplitude distribution function by averaging gamma amplitudes across these intervals. TGC values will then be determined by comparing the observed amplitude distribution against a uniform distribution (29):


$$\text{TGC}=\frac{\left[\left(\log\left(N\right)-\text{H}\left(P\right)\right)\right]}{\text{log}\left(N\right)}$$

Let *N* denote the quantity of phase bins, where *log (N)* denotes the entropy of a uniform distribution. *P* represents the relative amplitude distribution arranged by phase bins, and *H (P)* indicates the entropy of the *P* distribution, computed as follows:


$$H\left(P\right)=\;-\sum_{j=1}^{N}P\left(j\right)log\left[P\left(j\right)\right]$$

Increased coupling correlates with reduced entropy, leading to a higher TGC value. TGC is computed for each electrode, and then averaged across the right and left frontal electrodes (F7/8, F5/F6, F3/4, F1/2, and Fz). For this study, consistent with our previous work, we will use TGC as measured across target trials as target trials require a higher degree of ordering for correct performance, and thus, a higher level of TGC (**Figure 2B**) (29).

### Randomization

Participants will be assigned to one of the two treatment arms: tACS or sham-tACS. Given the importance to balance gender and baseline 2-back performance, and the relatively small sample size of the trial, we will use a dynamic allocation method, known as the covariate-adaptive randomization, to minimize the imbalance across the treatment arms (66). This method is advantageous over the conventional stratified randomization because it attains balance over more covariates with smaller sample size. It does increase the complexity of the design and the subsequent analyses. However, the current literature agrees that the benefit of this method overweighs its limitations in general (66). In detail, when a new participant enters the trial, we will calculate the degree of imbalance for each of the two possible assignments and the one that achieves the least total imbalance will be chosen with a higher probability. If there are assignments of the same least total imbalance, one of them will be chosen randomly. The degree of imbalance of the covariates of interest, gender and baseline 2-back performance, is calculated separately using standardized measure of distance across the two arms and the total imbalance is the sum of the imbalance scores.

### Intervention

#### Individualized transcranial alternating current stimulation

The alternating-current stimulation will be administered using M × N high-definition tACS stimulator (Soterix Medical). tACS will include multiple sintered 12 mm diameter Ag/AgCl electrodes that will be attached to high-definition plastic holders which will be embedded in a cap. The bipolar sinusoidal alternating current will be delivered to the PFC and temporal regions on both sides sequentially, randomly starting on the left or right side. The frequency of stimulation will be at the individually defined theta oscillation determined for each participant.

To fully individualize the approach adopted by Reinhart and Nguyen (35) in calculating theta frequency, we will use individuals’ own MRI alongside EEG. We will use the baseline N-back-EEG at 2-back condition and identify the endogenous theta peak frequency. To calculate the individualized tACS frequency, we conduct source analysis through a series of steps. Initially, after recording MRI data, we transform it to MNI coordinates. Then, we segment the MRI data into five layers: gray matter, white matter, cerebrospinal fluid (CSF), skull, and scalp. We set the conductivity of the segmented tissues and create a head model. This involves setting the conductivity values for gray matter to 0.33, white matter to 0.14, CSF to 1.79, skull to 0.01, and scalp to 0.43 (S/m). Subsequently, we will create a source model based on MNI template grid (35).

To perform source analysis, we apply the Linearly Constrained Minimum Variance (LCMV) method. LCMV estimates the activity of a specific source while concurrently suppressing contributions from other sources and noise (67). The resulting sources are then mapped to the Automated Anatomical Labeling (AAL) atlas, with a specific focus on identifying regions of interest in the superior frontal cortex and superior temporal regions.

In the source space, we will apply spectral decomposition across the 1 to 30 Hz frequency range with 0.1 Hz intervals for each trial. Data will be segmented to trials from -1,400 ms to 3,100 ms relative to letter onset, using the 2-back target and non-target letter as the stimulus. Using Morlet wavelets (constant center frequency ratio = 14 and cycles = 6), we will assess theta band synchronization between frontal and temporal regions. We will calculate phase-locking value in the source space between the left temporal cortex and left PFC, as well as the right temporal cortex and right PFC. We will examine the difference in phase-locking value between 2-back target and non-target trials that were responded to correctly within the time frame of 0 to response-time relative to the onset of the stimulus. We will focus on target trials as they require a higher degree of ordering for accurate performance compared to non-target trials, which rely on synchronization between temporal and frontal regions (29, 35). Within the theta band, we will identify the frequency with the maximum difference in mean phase-locking value between target and non-target trials on each side (left and right) for each participant. This frequency is then utilized as the target stimulation frequency for the corresponding side. Individualized frequency-tuned stimulation is administered with 0.5 Hz resolution, rounding up from 0.3 Hz (35).

During each session, stimulation on each side will last for 30 minutes with a 60-second ramp-up and ramp-down period. After stimulation is completed on one side, the scalp will be wiped clean, and a new cap will be used to minimize the shunting effect caused by excessive gel on the scalp. Sessions will occur 5 days a week for 2 weeks. During the sham-tACS procedure, the device will have a ramp up of 60 seconds to reach better tolerability, followed by an immediate decrease. At the end of the session, the device undergoes another 60-second ramp-up, followed by a ramp-down. This sham stimulation is designed to elicit the tingling sensation commonly experienced with active stimulation (35, 68, 69).

#### Optimizing the electrode montage

We will use SimNIBS (70, 71) for optimizing and determining the individualized electrode montage. The process begins with creating the head model based on the structural MRI image. Then, we will create a lead field matrix using default EEG cap in SimNIBS. We will optimize the current and location of stimulation electrodes (i.e., electrode montage) to achieve the maximum electrical field in the 3 mm radios spheres centered at targeted frontal and temporal regions of each hemisphere, specifically the superior frontal gyrus dorsolateral (right and left) and superior temporal gyrus (right and left).

**Figure 3:**
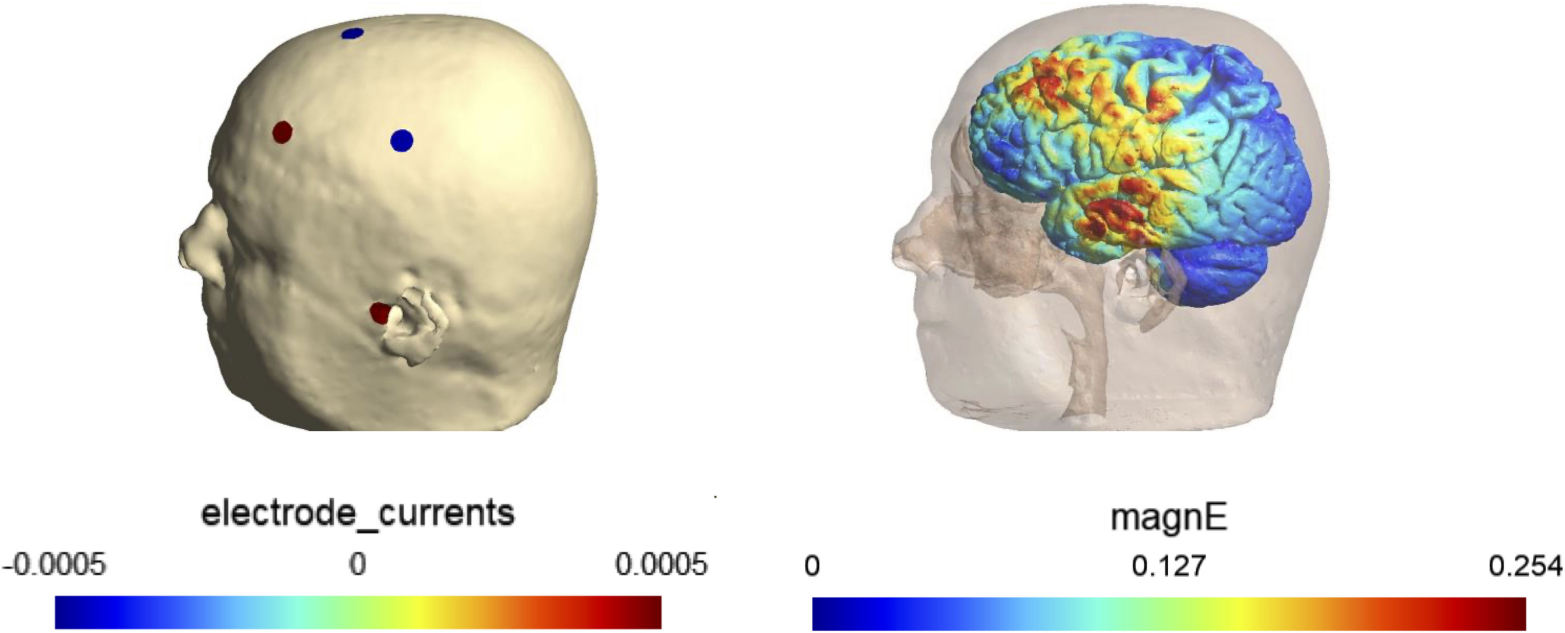
An example of an individualized montage and simulated electric field.

For safety considerations, we will impose constraints on the stimulation parameters. The maximum total current is set to 1 mA (baseline to peak), and the current through any single electrode will not exceed 0.5 mA. Additionally, no more than eight electrodes will be active during the stimulation. The electric field at targets will be calculated using current flow simulation based on the optimized electrode montage. An example of an individualized montage for stimulating left PFC and temporal regions is shown in **Figure 3**.

The montage is designed to maximize electric filed in PFC and temporal targets based on the individual’s MRI. The electrode montage is shown on the left and the simulated electric field is shown on the right.

### Blinding

This study will be conducted under triple-blind conditions (1): Participants will be blind to what intervention they will receive (active or sham) (2); the research assistant delivering the 10-session course will be blind to group assignment; and (3) the research assistant conducting the N-back-EEG assessments and cognitive assessments at baseline and follow-up will also be blind to group assignment. We will assess the participants’ expectancy of treatment outcome using Stanford Expectations of Treatment Scale (SETS) as it can affect their response to intervention (72). It has been shown that participants’ belief can also affect the intervention outcome (73). To evaluate the integrity of the blinding, we will ask participants, interventionists, assessors, and the Principal Investigator, to guess which intervention the participant received, both after the first intervention session and at the completion of the study.

### Analytic strategy and power analysis

#### Sample size determination

This study is designed to generate pilot data for future studies. Thus, no *a priori* data is used to calculate the sample size. However, with the proposed sample size, we anticipate reliable estimates of recruitment (15.5% margin of error) and retention rate (17.5% margin of error) (Objective 1). For Objectives 2 and 3, we will have 46% power to detect a large treatment effect (Cohen’s d = 1.0) and the minimum detectable effect size to attain 80% power is 1.5. These values are consistent with the pilot nature of the study. We have taken into account a 20% attrition rate and used a 95% confidence interval or two-tailed tests with 0.05 significance level in the power analysis.

#### Statistical methods

Variables will be first subjected to descriptive analyses. Non-normal data will be transformed, or analyzed using nonparametric procedures. Following intent-to-treat principles, all randomized participants will be considered in the analyses. For Objective 1, we will primarily conduct descriptive analysis to estimate recruitment rate and retention rate using 95% confidence intervals. For Objectives 2 and 3, linear regression will be used to examine the association between TGC engagement and changes in working memory following EEG and MRI-guided individualized tACS courses. Covariates, including demographics and baseline outcome measures, will be incorporated into the model in sensitivity analyses with the understanding that the proposed sample size does not support complex analysis. Full information maximum likelihood method will be employed to account for potential bias incurred by missing data. All missing cases and their reasons will be documented.

## Discussion

In this section, we discuss the rationale behind the key components of our study design.

### Rationale for tACS

tACS involves delivering an alternating sinusoidal current of approximately 0.5–1 mA baseline-to-peak at a specific frequency based on the brain oscillation the tACS aims to entrain. A usual tACS session lasts between 20 to 60 min. Research has shown that standard tACS delivered at gamma frequency and to the dorsolateral PFC improve recognition in episodic memory (74) and retrieval (75) in healthy young adults. Standard tACS delivered at the theta frequency and to the temporoparietal regions has also been shown to improve long-term memory recognition in healthy younger adults (76, 77) and associative learning in healthy older adults (78). This evidence underscores the potential of tACS as a powerful tool for modulating cognitive functions through targeted brain stimulation.

Few studies have investigated the effects of tACS in patients with AD or MCI. In one study, 30 individuals with multi-domain amnestic MCI were randomized to either home-based frontal theta tACS (6 Hz, current of 1.5 mA baseline-to-peak) combined with cognitive control training (CCT) or control tACS (1 Hz) for 8 sessions. This study reported high tolerability and adherence, along with improvements in attention in the group receiving theta tACS + CCT compared to the control group (79). Another study examined gamma tACS (40 Hz) on individuals (N=13) with amnestic MCI, targeting 8 electrodes (F7, F8, FT7, FT8, T7, T8, P7, and P8; 10–20 EEG system; total current of 1.6 mA baseline-to-peak) over 5 days in the first week and one day per week for the next 3 weeks while performing cognitive tasks. This study found gamma tACS to be feasible in this population and reported improvement in episodic memory, although no changes were observed in fluid biomarkers. Notably, improvements in episodic memory were positively associated with the induced electric field based on current flow modeling (80). In AD participants, a small pilot study (N=8) showed that home-based multi-channel tACS at 40 Hz (6 electrodes and current from each electrode was below 2.0 mA) targeting the left angular gyrus led to improved memory performance without changes in the MoCA score. This study used current flow modeling to optimize the tACS montage for targeting the left angular gyrus, but it used a standard brain model and the montage was not individualized (“non-individualized optimized stimulation”) (81). A larger randomized double-blind study used gamma tACS (current of 1.5 mA baseline-to-peak) in AD participants (N=60). The study found a significant effect of tACS on Rey Auditory Verbal Learning (RAVLT) and face-name association scores in the active group compared to sham tACS group. Using current flow modeling, this study also demonstrated that the induced electric field was associated with clinical outcomes (82). In one non-controlled, open-label, small pilot study (N = 17), 11 participants with mild-to-moderate dementia received tACS (40Hz, current of 0.75 baseline-to-peak) over the left dorsolateral PFC for 30 min twice a day combined with cognitive training sessions, 2 sessions/day for 5 days/week for 4 weeks (83). Compared to those 6 participants who received only training sessions, these 11 participants experienced lasting improvements in memory as assessed at a 1-month follow-up post intervention.

Despite these promising findings, all studies have utilized the same montage and frequency for all participants, which may not be optimal given structural and functional differences between individuals (38, 84). There is promising evidence that individualized electrode montage informed by participants’ structural data, can enhance the efficacy of tACS by addressing inter-individual variability in its effect (85).

*tACS and TGC:* No study to date has specifically assessed the effect of standard or individualized tACS on TGC and cognitive function in AD or MCI. However, frequency-individualized tACS has been shown to engage TGC and, through this engagement, to enhance working memory in older healthy adults (35). In this study, as expected, young healthy adults demonstrated better working memory than older adults at baseline (i.e., before tACS). In addition, and unlike older adults, they demonstrated theta synchronization between the left PFC and the left temporal cortex during the working memory task as well as TGC across the left temporal electrodes. In contrast, older adults did not exhibit theta synchronization nor TGC. Following frequency-individualized tACS, older adults’ performance improved to a level that became no different from that of younger adults. Further, PFC-temporal theta phase synchronization improved in older adults. Most importantly, TGC emerged over the left temporal electrodes in older adults and the strength of TGC was predictive of their performance on the working memory task post-tACS (35).

Taken together, the current literature suggests that tACS can enhance cognitive function in both healthy adults and those with AD and MCI. It also suggests that when tACS is individualized to person-specific theta frequency (frequency-individualized stimulation) and target PFC-temporal connections, it can robustly enhance TGC and, in turn, improve working memory. **Table 2** summarizes the ongoing tACS studies in MCI and AD populations.

**Table 2:** Ongoing tACS studies in MCI and AD population.

PI (Location)	ClinicalTrials.gov Identifier	Condition (N)	Intervention	Primary Outcome	Difference from Current Proposal
Study Title: Gamma Induction for Alzheimer’s Disease					
Santarnecchi (Boston, USA)	NCT03880240	AD MCI (55)	tACS vs. Sham	PET amyloid burden PET tau deposition Gamma activity ADAS-Cog	Different biological target
Study Title: Individual Closed-Loop Neuromodulation Therapy for Alzheimer’s Disease					
Camprodon (Boston, USA)	NCT05904132	AD-MCI (70)	tACS vs, Sham	Cognitive performance Changes in the power of entrainment of natural gamma rhythms	Different primary biological target
Study Title: Memory Enhancement Using Transcranial Alternating Current Stimulation (Memento)					
Haan (Amsterdam, Netherland)	NCT06202872	AD-MCI (30)	tACS vs, Sham	Rey Auditory Verbal Learning test Face-Name Association Task	No primary biological target
Study Title: Understanding Brain Mechanisms Involved in Autobiographical Memory					
Michel (Geneva, Switzerland)	NCT05710549	MCI Old Healthy Young Healthy (120)	tACS vs. Sham	Spatiotemporal dynamic changes measured with electroencephalography (hdEEG) Cognitive Assessment	Different primary biological target
Study Title: The Treatment of High Intensity Transcranial Current Stimulation for Alzheimer’s Disease					
Shi (Anhui, China)	NCT06380725	AD (60)	tACS vs. Sham	Cognitive appraisal Global Cognitive appraisal Psychobehavioral assessment	No primary biological target
Study Title: Speed of Processing (SoP) Training Plus α-tACS (aMCIUp)					
Jorge Leite (Porto, Portugal)	NCT05198726	MCI (327)	tACS vs, Sham	Useful Field of View (UFOV)	No primary biological target
Study Title: 40Hz tACS in Treating Cognitive Function and Modulating Neurophysiology of Patients With Alzheimer’s Disease					
Chu (Kaohsiung, Taiwan)	NCT05723172	AD (48)	tACS vs, Sham	ADAS-Cog	No primary biological target
Study Title: Improvement of Memory in Mild Cognitive Impairment					
Michel (Geneva, Switzerland))	NCT05708001	MCI (80)	tACS vs, Sham	MoCA	No primary biological target
Study Title: Clinical Trial Through Combined tACS Therapy in Patients With Mild Cognitive Impairment					
Billeke (Chile)	NCT05291208	MCI (62)	tACS and Cognitive Training Program vs. Sham	Prefrontal theta oscillation activity	Different primary biological target
Study Title: Non-invasive Brain Stimulation for Cognitive and Motor Dysfunction in Dementia (ACDCStim)					
Pascual-Leone (Boston, USA)	NCT05661084	MCI Mild dementia (144)	tACS + tDCS, tACS+Sham tDCS, Sham tACS, tDCS, Sham	Change in Rey Auditory Verbal Learning Test (RAVLT) Total Recall Change in Dual Task Cost to Gait	No primary biological target
Study Title: Novel, Individualized Brain Stimulation, Network-based Approaches to Improve Cognition in Healthy Seniors and Patients With MCI					
Hummel, Rektorova (Czechia)	NCT04986787	MCI Healthy Aging (160)	tACS and TMS vs. Sham	Working memory	No primary biological target
Study Title: Modulating Cortical Dynamics of Dual-task Standing in MCI					
Kahya (Boston, USA)	NCT05680701	MCI Healthy Aging (60)	tACS vs. Sham	EEG alpha-band power Postural sway speed	Different primary biological target
Study Title: Non-invasive Neurostimulation as a Tool for Diagnostics and Management for Neurodegenerative Diseases					
Solje (Kuopio, Finland)	NCT05326750	Alzheimer Disease Frontotemporal Dementia Dementia With Lewy Bodies (200)	tACS vs. Sham	Changes in Rey Auditory Verbal Learning Test score Changes in Rey Auditory Verbal Learning Test, recognition Changes in orientation to time and place (MMSE questions 1-10) Changes in Trail Making A & B tests Changes in INECO Frontal Screening test Changes in phonemic fluency	No primary biological target
Study Title: Effect of Transcranial Alternating Current Stimulation(tACS) for Early Alzheimer’s Disease					
KAI (Anhui, China)	NCT06565143	Early Alzheimer’s Disease (40)	tACS vs. Sham	ADAS-Cog	No primary biological target

clinicaltrials.gov database was reviewed until December 13, 2024 using search terms of Alzheimer Disease, MCI and tACS.

### Supporting data for working memory and TGC

Several groups, including ours, have demonstrated that TGC is associated with working memory in healthy (29, 86, 87) and clinical populations, including those with MCI (32). In patients with MCI, we found that TGC during a working memory task is impaired compared to healthy older adults even though these MCI patients were minimally (and not significantly) impaired on performance of the working memory task (32). These findings suggest that TGC in MCI patients is linked to cognitive compensation, which would have to decrease below a specific threshold before behavioral impairment occurs. By enhancing TGC, our goal is to enhance cognitive compensation and, in turn, prevent cognitive decline in patients with MCI.

Further, TGC is not only associated with working memory performance during the execution of the N-back task, but also with performance on other working memory and executive function tasks that require PFC-supported manipulation and context-based ordering of information (30). Importantly, the associations between TGC and performance on these other tasks were present even when TGC and the tasks were administered several weeks apart. This provides evidence for a stable and trait-type relationship between TGC and PFC function (30). We have also demonstrated in a longitudinal study in healthy older individuals that changes in TGC are associated with changes in working memory over a 12-week of follow-up period (88). Finally, we have shown that TGC is associated with working memory performance across different age groups, showing its relevance irrespective of aging (87).

In addition to these cross-sectional and longitudinal studies, intervention studies further support the role of TGC in working memory. In one study, frequency-individualized tACS enhanced TGC in healthy older individuals and, through this enhancement, improved their working memory (35). Another study has shown that using peak-coupled theta–gamma cross-frequency tACS targeting the dorsolateral PFC improves 2-back task in healthy older adults (33). In another study, transcranial direct current stimulation (tDCS) was shown to enhance working memory via TGC enhancement in a group of healthy younger adults (89). Taken together, these studies support that TGC is a promising target to engage for the enhancement of working memory and PFC function, particularly in individuals with MCI.

### Support for PFC-hippocampus connections role in working memory and TGC

PFC-hippocampus pathways are well established (14, 90). One key bidirectional connection between the PFC and the hippocampus is via the perirhinal and lateral entorhinal cortices (91, 92). Via this connection, specific item representations (e.g., representations of the new letters during the N-back task) are supported for processing (93, 94). Another key bidirectional connection is via the thalamic nucleus reuniens (Re) (95, 96). The Re is thought to couple the hippocampus and the PFC by synchronizing the two areas (97–99) supporting the transfer of these specific items representations between the hippocampus and PFC for processing. Through these connections, excitatory glutamatergic pyramidal neurons from the hippocampus project and terminate on principle neurons (100, 101) and GABAergic interneurons in the PFC (102, 103). Oscillatory synchrony emerges and the PFC and the hippocampus are coupled, and, in turn, operate as a system in which the PFC receives information that is salient to the current context provided by the hippocampus (104–106).

The hippocampus has been shown to entrain PFC theta oscillations (24, 25),. Over time and as memories become long-term, the interaction between PFC and the hippocampus reverses such as the PFC starts leading the hippocampus in theta oscillations to retrieve long-term memories (107).

In mice, PFC-hippocampus connections have also been shown to support TGC between hippocampal theta and PFC gamma oscillations (31, 108). In one of these studies, these connections have also been shown to support working memory by supporting TGC. Within the mouse PFC, local-field potential recordings demonstrated TGC (31). Further, gamma oscillations within the PFC were coupled to theta oscillations within the hippocampus (31). Interestingly, in a mutant mouse model that demonstrates impairment in working memory, there was an increase in strength of TGC between hippocampal theta and PFC gamma oscillations when these mice performed correctly on the working memory task. By contrast, no changes were observed in local PFC TGC - i.e., TGC between PFC gamma and PFC theta oscillations (31). In addition, firing of PFC neurons was phase-locked to PFC local gamma oscillations that were coupled with hippocampal theta oscillations. These findings strongly support the PFC-hippocampus connection role in mediating a compensatory cognitive mechanism via TGC, i.e., the mechanism that we propose to optimize in our study.

Human studies also support the role of the PFC-hippocampus connections in TGC. In one study, adults with acute depression who received repetitive Transcranial Magnetic Stimulation (rTMS) to the left PFC and experienced reduced clinical symptoms, increased left hippocampal volume, and enhanced TGC over the left central area. Further, changes in TGC were correlated with changes in hippocampal volume (109). Additionally, using intracranial EEG recordings in presurgical epilepsy patients, one study showed an association between TGC within the hippocampus and memory performance (110). Recently, another intracranial EEG study in patients with drug-resistance epilepsy highlighted the role of TGC in coordinating frontal cognitive control and maintenance of information in hippocampus during working memory (34).

## Limitations and future directions

There are several areas that warrant further investigation in future work. First, although the use of a sham control condition allows us to assess the feasibility and tolerability of EEG and MRI-guided tACS, it does not fully disentangle the contributions of transcranial versus sensory stimulation. Prior research suggests that gamma tACS, when phase-locked to the trough of theta tACS, does not modulate TGC (111). Building on our findings, future studies focused specifically on underlying mechanisms could incorporate such waveforms to examine their impact on frontal and temporal synchronization and their potential as a control condition to complement or replace sham.

Second, our study employs individualized targeting based on EEG and MRI to optimize specificity and potential efficacy. However, we recognize that in some clinical settings, such methods may not be feasible due to cost or limited access to imaging technologies. Future work could explore whether standardized targeting strategies offer similar benefits to enhance scalability for broader clinical use.

Third, while our stimulation parameters were carefully selected based on modeling, current protocol does not include systematic manipulation of stimulation frequency or anatomical location. Future studies may build on our results by exploring how variations in these parameters influence outcomes.

In conclusion, the overall goal of this study is to use EEG and MRI-guided individualized tACS to strengthen the bidirectional connections between the PFC and the hippocampus. We will stimulate these two regions bilaterally, directly to the PFC and indirectly to the temporal cortices, to optimize their connectivity, enhance TGC, and cognitive performance. The ultimate goal is to enhance overall cognitive performance and mitigate the risk of neurodegenerative processes.
